# Research Progress on RET Fusion in Non-Small-Cell Lung Cancer

**DOI:** 10.3389/fonc.2022.894214

**Published:** 2022-05-30

**Authors:** Lu Zhao, Qingyun Mei, Yongchao Yu, Na Wang, Dou Zhang, Dongying Liao, Jinhui Zuo, Hongxia Xie, Yingjie Jia, Fanming Kong

**Affiliations:** ^1^ Department of Oncology, First Teaching Hospital of Tianjin University of Traditional Chinese Medicine, Tianjin, China; ^2^ National Clinical Research Center for Chinese Medicine Acupuncture and Moxibustion, Tianjin, China

**Keywords:** NSCLC, RET fusion, targeted therapy, drug resistance, immunotherapy

## Abstract

Great progress has been made in the treatment of driver gene-positive Non- Small Cell Lung Cancer (NSCLC) in recent years. RET fusion was seen in 0.7% to 2% of NSCLC and was associated with younger age and never-smoker status. The pralsetinib and selpercatinib for RET fusion NSCLC was recommended by the 2021 NSCLC treatment guidelines. This review outlines the research progress in the treatment of RET fusion NSCLC, identifies current challenges and describes proposals for improving the outlook for these patients.

## Introduction

Lung cancer ranks second among malignant tumors in the world, the long-term outcome is still poor (1). Non-small cell lung cancer (NSCLC) accounts for approximately 80% to 85% (2). Most NSCLC patients were diagnosed at an advanced stage, and platinum-containing combination chemotherapy was the main treatment, but the five-year survival rate was only approximately 15% (3). The incidence of RET fusion in NSCLC was 0.7% - 2% and was associated with younger age and never-smoker status (4). Among the 12 identified fusion genes, the most common partner genes were KIF5B, CCDC6, and NCOA4 (5).

RET, acting as a proto-oncogene, was first identified in NIH/3T3 cells from transformed mice in 1985 (6). It was located on chromosome 10q11.2 and contained 21 exons with a full length of 60 kb (7). It encodes the RET transmembrane receptor kinase, which is needed for proliferation, differentiation, migration (8–10). RET fusion could activate the downstream PI3K/AKT, RAS/MAPK, and JAK/STAT pathways, which further promoted tumor proliferation, differentiation, migration. The clinicopathological characteristics of RET fusion NSCLC were as follows: younger age (60 years old), never smoking, adenocarcinoma, smaller volume (≤3 cm), lymph node metastasis, low differentiation (11, 12). It was less likely to have other oncogenic driver genes together, suggesting its own oncogenic driver potential. Moreover, RET fusion was also associated with a high risk of metastasis to the brain (13).

Currently, RET-selective TKIs such as pralsetinib and selpercatinib were the main treatments for RET fusion NSCLC patients. The acquired drug resistance was found in the clinical. And the platinum-containing chemotherapy or Immune-Checkpoint Inhibitors(ICIs) was explored as therapic choice after drug resistance. This article systematically reviewed the recent advances in the treatments for RET fusion NSCLC, provided current challenges and described proposals for improving the future clinical management of the disease.

## RET-Selective TKIs

### Selpercatinib(Retevmo)

Selpercatinib was a highly selective RET inhibitor that could block the adenosine triphosphate binding site of RET receptor tyrosine kinase (14). Because of good properties in target specificity, tolerability, and intracranial efficacy, Selpercatinib became a new valid option for many European patients (15). A total of 105 RET fusion NSCLC patients enrolled in the phase 1/2 LIBRETTO - 001 trial received the Selpercatinib treatment had promising ORR(64%) and mDoR(17 months), and the mPFS of these patients was 16 months (16–18) (**Table 1**). Based on the anti-tumor efficacy and safety, Selpercatinib was approved by FDA for RET fusion NSCLC on May 8, 2020. In 2021, Selpercatinib was approved by the EMA and Swiss-Medic for second line or posterior line therapy. The clinical trial LIBRETTO-321 was conducted to evaluate the efficacy of Selpercatinib for Chinese RET fusion NSCLC patients. The ORR was 61.1% in the Selpercatinib treated population. 90% of the patients remained in continuous remission after 6 months (19). This study indicated that Selpercatinib was also a promising therapeutic option for Chinese RET fusion NSCLC patients. Currently, two large clinical studies, namely, LIBRETTO-121 and LIBRETTO-431 are ongoing to explore the efficacy of Selpercatinib in advanced solid tumors (20, 21).

**Table 1:** MKI and TKI in RET fusion NSCLC.

NCT-number	Trial group	Trial Design	primary end points	Secondary end point	Adverse reactions greater than or equal to grade 3
Pralsetinib (BLU-667) (NCT03037385)	233 patients with RET fusion-positive NSCLC	**The phase 1** dose escalation part of the trial determined the maximum tolerated dose **The phase 2** dose expansion part evaluated the safety and activity of pralsetinib in multiple expansion groups	mPFS: 9.1months OS: <13.6months The tolerated dose was 400mg	RR:53% ORR:33%	Gr3-4 treatment-related Pralsetinib=48%
Selpercatinib (LOXO-292) (NCT03157128)	253 patients with RET fusion-positive NSCLC	Selpercatinib, 160 mg orally twice daily, was administered in consecutive 28-day cycles until disease progression	ORR:64% mPFS:16months	mDOR:17months	Gr3-4 treatment-related Selpercatinib=NR
Cabozantinib (NCT01639508)	26 patients with RET fusion-positive NSCLC	Patients were given 60 mg of cabozantinib orally per day until disease progression	mPFS:5.5months mOS:9.9months	RR:28%	Gr3-4 treatment-related cabozantinib=46%
Vandetanib (UMIN000010095)	19 patients with RET fusion-positive NSCLC	Patients were continuously received 300 mg of oral vandetanib daily until disease progression	mPFS:6.5months mOS:13.5months	ORR:47.4% DCR: 89.5%	Gr3-4 treatment-related Vandetanib=84.2%

### Pralsetinib(Gavreto)

Pralsetinib was the second selective RET inhibitor with highly potent and selective for wild-type RET, RET fusion (including the most common KIF5B-RET and CCDC6-RET), and mutations (RET V804 L, RET V804 M, and RET M918T) (22). The clinical activity and safety of Pralsetinib was investigated by the ARROW study(Global multicentric single-arm phase I/II trial) (23). Based on the result of this study, Pralsetinib was approved as first-line or post-line treatment for RET fusion NSCLC by FDA in September 2020 (24, 25). Updated data reported by the American Society of Clinical Oncology (ASCO) in 2021 showed that the ORR was 17.1 months, the CR was 6%, and the mPFS was 16.5 months (n=136). Nine patients with measurable brain metastases all showed an intracranial reduction to a certain extent (intracranial response rate 56%, intracranial CR 33%) (**Table 1**). As the excellent efficacy and low off-target toxicity in RET cancer patients, Pralsetinib was also approved by China’s State Food and Drug Administration (NMPA) in March, 2021 (26). This is the first RET inhibitor approved in China and is of great significance (27–29).

## Nonselective Multitargeted TKIs

### Cabozantinib

Cabozantinib was a multikinase inhibitor against RET, VEGFR2, MET, ROS1, AXL, c-KIT, TIE2, and FLT3 (30). The clinical application of Cabozantinib was limited due to multi-target inhibition and the off-target effects (**Table 1**). A 2016 phase II clinical study consisting of 26 patients with RET fusion showed a mPFS of 5.5 months, a mOS of 9.9 months, and overall efficiency of only 28% (31). The overall efficiency of Cabozantinib for RET fusion patients was significantly lower than ALK/ROS1 gene fusion and EGFR mutation patients (32). Other results from the global multicenter registry also showed unsatisfactory clinical effect and highly incidence of grade 3/4 adverse effects (33–36).

### Vandetanib

Vandetanib was a multikinase inhibitor that inhibits VEGFR, EGFR, and RET (37). In the LURET phase II study, the enrolled 19 Japanese RET fusion patients received the Vandetanib treatment, the median PFS was 4.7 months, the median OS was 11.1 months, and the overall survival rate at 12 months was 52.6%. Eleven patients (57.9%) had adverse events leading to a dose reduction (38). Another phase II study explored the efficacy of Vandetanib in Korean patients with metastatic or recurrent RET fusion NSCLC. The study showed that the median PFS was 4.5 months, the median OS was 11.6 months (**Table 1**). The most common grade 3 AEs were hypertension (17%), a prolonged QTc interval (11%), and transaminitis (6%) (39).

### Other MKIs

Other MKIs had limited clinical data, and since the development of selective TKIs, they may have less attention. In 2017, a global multicenter RET registry study (GLORY) retrospectively explored the efficacy of RET fusion NSCLC patients using varieties of MKIs. At the time of the analysis, only 15% of the patients were continuing their treatment. This indicated that most patients did not benefit from these MKIs. As **Table 2** showed, Lenvatinib only had an ORR of 16% in phase 2 multicenter trial (46, 47). The optimal response of sorafenib was SD, with no objective response (48). Sunitinib has a similar TKI activity profile to sorafenib but showed slightly better activity with a DCR of 55% (49). Other experimental drugs with Anti-Ret activity but limited preclinical data include apatinib, AD80, and dovitinib (50). However, the clinical efficacy has not yet been demonstrated.

**Table 2:** ICIs in RET fusion NSCLC.

Reference	Characteristics	ORR (%)	mPFS (months)	mOS (months)
Dudnik E et al. (40)	RET fusion (n=4)	0	3	14.9
Guisier F et al. (41)	RET fusion (n=9)	37.5	7.6	Not available
Hegde A et al. (42)	RET fusion (n=16)	Not available	3.4	Not available
Lee J et al. (43)	RET fusion (n=13)	7.7	2.1	12.4
Lu C et al. (12)	RET fusion (n=10)	20	2.5	Not available
Mazières J et al. (44)	RET fusion (n=16)	6	2.1	21.3
Offin M et al. (45)	RET fusion (n=13)	Not available	3.4	Not available

## Chemotherapy

Until the appearance of specific RET inhibitors, chemotherapy was still the primary treatment for RET fusion NSCLC. Some studies showed that these patients were sensitive to pemetrexed-based regimens. In the GLORY global study, 108 advanced NSCLC patients with RET fusion received first-line chemotherapy, the median PFS was 6.6 and 7.8 months, while the median OS was 23.6, 24.8months, respectively (45, 51). In summary, chemotherapy could bring some clinical benefits for RET fusion NSCLC. Before the clinical accessibility or inapplicable of targeted drugs, platinum-based regimens was one of the treatment options for RET fusion NSCLC patients.

## ICIs

Immune checkpoint inhibitors (ICIs) have become the standard treatment for driver gene-negative metastatic NSCLC. However, it proved poorly effective in patients with positive driver gene mutations, such as EGFR and ALK positive patients. The efficacy of ICIs in RET fusion NSCLC was insufficiency. The existing studies were summarized to assess the efficacy of ICIs treatment in RET fusion NSCLC patients.

### ICIs as First-Line Treatment

In a single-center retrospective study, 4 RET fusion NSCLC patients and 1 RET-mutant NSCLC patient received ICIs treatment, and the mPFS and mOS were 3.0 months and 14.9 months, respectively (40). In a single-center retrospective study of Korea, the efficacy of Vandetanib was compared to chemotherapy in 59 RET fusion NSCLC patients, the ORR was 15.8% in the Vandetanib group, while 63% in chemotherapy group. For the ICIs group, the ORR was 7.7%, the PFS was 2.1 months, and the OS was 12.4 months (43). In contrast, another retrospective analysis of 45 Chinese patients showed no significant difference in PFS obtained with MKI *vs.* chemotherapy *vs.* ICIs treatment (3.8 *vs.* 3.5 *vs.* 2.5 months), the 10 evaluable patients treated with ICIs had an ORR of 20% (44). The difference between Chinese and Korean patients indicated that ICIs treatment remained controversial in RET fusion NSCLC patients (**Table 2**).

ICIs combined with chemotherapy therapy could be a choice for RET fusion NSCLC patients. In a retrospective real-world study, 12 patients with RET fusion NSCLC were treated with palivizumab combined with chemotherapy, the ORR was 58%, mPFS was 5.4 months, and OS was 19 months (52). Subsequently, Hess, et al. have verified the clinical benefit of the above chemotherapy combined with ICIs in 9 RET fusion NSCLC patients. The ORR was 66% and mPFS 6.6 months (**Table 2**) (41, 53, 54).

### ICIs as Post-Line Treatment

In a multicenter retrospective study, 11 RET fusion NSCLC patients received ICIs in the post-line treatment. The DCR was up to 60% (41, 55). This result suggested that some patients in second-line and post-line treatment could benefit from ICIs. The comprehensive guidelines recommend that ICIs as a second-line treatment for RET fusion NSCLC patients (**Table 2**).

## Combined Therapy

Vandetanib in combination with everolimus brought about remission in all six RET fusion-positive NSCLCs. It also showed antitumor activity in refractory cases with cabozantinib and brain metastasis. Besides MET amplification was detected in four patients in the LIBRETTO-001 study, combination treatment with the selpercatinib+MET/ALK/ROS1 inhibitor crizotinib also showed clinical activity and good tolerability (42). In summary, combined treatment methods may provide clinical benefit to patients with RET fusion NSCLC, but their safety needs to be further verified.

## Drug Resistance

### Mechanisms of On-Target Drug Resistance

It was an intratarget kinase-acquired resistance that dynamically evolves under kinase inhibitor selection pressure, making the kinase continuously activated under medication conditions. Gatekeeper mutations and solvent-front mutations were included. It has been reported that resistance mechanisms in MKIs include RET V804 M gatekeeper mutations and RET S904F (55). The selective RET inhibitor Selpercatinib and Pralsetinib induced a pre-lytic mutation (G810A/S) (56). It also demonstrated that it increased kinase activity and conferred resistance through allosteric effects. AXL overexpression and RAS mutations were subsequently reported in two RET fusion-positive cell lines resistant to multikinase inhibitors (22). Selective RET inhibitors have been designed to overcome gatekeeper mutations. The concurrent RET V804M gatekeeper mutation was associated with a G810 resolute mutation in an NSCLC patient.

### Mechanisms of Off-Target Drug Resistance

The mechanisms of off-target resistance included the reactivation of different intracellular pathways, bypassing targeted receptor kinase-mediated signals. MET dependence has been reported as a recurrent and potential targeting mechanism for resistance to Selpercatinib and Pralsetinib (19, 57). A study that analyzed 20 RET fusion NSCLC patients who were resistant to Selpercatinib and Pralsetinib, the study found there were 3 (15%) MET amplification, 2 (10%) solvent G810C/S resistance, and 1 (5%) KRAS amplification. Recently, BRAF V600E mutation following Selpercatinib treatment was reported in KIF5B-RET fusion NSCLC patient.

### Next-Generation of RET-TKIs

Currently, the next generation of RET-TKIs is under exploration. TPX-0046 is an efficient, selective, new generation of RET/SRC inhibitors. TPX-0046 is different in both structure and mechanism from Pralsetinib (Gavreto) and Selpercatinib (Retevmo). At present, TPX-0046 has been confirmed to have a clinical effect in patients (13, 58). BOS172738 is a targeted inhibitor of aberrant mutations in RET. A phase I clinical trial of BOS172738 reported that BOS172738 showed good safety for long-term administration. The overall efficacy ORR assessed by the investigator was 33% (n=18/54), and the NSCLC cohort ORR was 33% (n=10/30) (18, 59). Currently, multiple clinical trials are being conducted, including LIBRETTO-431, LIBRETTO-531, NCT04211337, and NCT03780517 (60).

## Summary and Prospects

The RET fusion NSCLC patients were advanced at diagnosis and had a poor prognosis. The highly selective RET inhibitors Selpercatinib and Pralsetinib provided new options for the treatment of RET fusion NSCLC patients. The occurrence of acquired resistance deserve attention, the next generation of RET TKIs or the novel therapeutic model were urgently need to be explored.

## Author Contributions

LZ and QM contributed equally to this work. All authors contributed to the article and approved the submitted version.

## Funding

This work is supported by the National Natural Science Foundation of China (No. 81403220), Tianjin Health and family planning-high level talent selection and training project, National key research and development (R&D) plan (2018YFC1707400).

## Conflict of Interest

The authors declare that the research was conducted in the absence of any commercial or financial relationships that could be construed as a potential conflict of interest.

## Publisher’s Note

All claims expressed in this article are solely those of the authors and do not necessarily represent those of their affiliated organizations, or those of the publisher, the editors and the reviewers. Any product that may be evaluated in this article, or claim that may be made by its manufacturer, is not guaranteed or endorsed by the publisher.

## References

[1] Siegel RL Miller KD Jemal A. Cancer Statistics, 2020. CA: Cancer J Clin (2020) 70(1):7–30. doi: 10.3322/caac.21590 31912902

[2] Ferlay J Colombet M Soerjomataram I Parkin DM Piñeros M Znaor A. Cancer Statistics for the Year 2020: An Overview. Int J Cancer (2021). doi: 10.1002/ijc.33588 33818764

[3] Cong XF Yang L Chen C Liu Z. Kif5b-Ret Fusion Gene and Its Correlation With Clinicopathological and Prognostic Features in Lung Cancer: A Meta-Analysis. OncoTargets Ther (2019) 12:4533–42. doi: 10.2147/ott.S186361 PMC6568188 31289444

[4] Myrand SP. A Novel Oncogenic Ret Fusion Variant in Nsclc: Relch-Ret. J Thorac oncol: Off Publ Int Assoc Study Lung Cancer (2021) 16(10):e95. doi: 10.1016/j.jtho.2020.12.018 34561049

[5] Schubert L Le AT Estrada-Bernal A Doak AE Yoo M Ferrara SE. Novel Human-Derived Ret Fusion Nsclc Cell Lines Have Heterogeneous Responses to Ret Inhibitors and Differential Regulation of Downstream Signaling. Mol Pharmacol (2021) 99(6):435–47. doi: 10.1124/molpharm.120.000207 PMC11033948 33795352

[6] Takahashi M Cooper GM. Ret Transforming Gene Encodes a Fusion Protein Homologous to Tyrosine Kinases. Mol Cell Biol (1987) 7(4):1378–85. doi: 10.1128/mcb.7.4.1378-1385.1987 PMC365224 3037315

[7] Takeuchi K. Discovery Stories of Ret Fusions in Lung Cancer: A Mini-Review. Front Physiol (2019) 10:216. doi: 10.3389/fphys.2019.00216 30941048 PMC6433883

[8] Cascetta P Sforza V Manzo A Carillio G Palumbo G Esposito G. Ret Inhibitors in Non-Small-Cell Lung Cancer. Cancers (2021) 13(17):4415. doi: 10.3390/cancers13174415 34503226 PMC8431193

[9] Drusbosky LM Rodriguez E Dawar R Ikpeazu CV. Therapeutic Strategies in Ret Gene Rearranged Non-Small Cell Lung Cancer. J Hematol Oncol (2021) 14(1):50. doi: 10.1186/s13045-021-01063-9 33771190 PMC7995721

[10] Bronte G Ulivi P Verlicchi A Cravero P Delmonte A Crinò L. Targeting Ret-Rearranged Non-Small-Cell Lung Cancer: Future Prospects. Lung Cancer (Auckland NZ) (2019) 10:27–36. doi: 10.2147/lctt.S192830 PMC6433115 30962732

[11] Zhang K Chen H Wang Y Yang L Zhou C Yin W. Clinical Characteristics and Molecular Patterns of Ret-Rearranged Lung Cancer in Chinese Patients. Oncol Res (2019) 27(5):575–82. doi: 10.3727/096504018x15344979253618 PMC7848427 30131091

[12] Lu C Zhou Q. Diagnostics, Therapeutics and Ret Inhibitor Resistance for Ret Fusion-Positive Non-Small Cell Lung Cancers and Future Perspectives. Cancer Treat Rev (2021) 96:102153. doi: 10.1016/j.ctrv.2021.102153 33773204

[13] Rebuzzi SE Zullo L Rossi G Grassi M Murianni V Tagliamento M. Novel Emerging Molecular Targets in Non-Small Cell Lung Cancer. Int J Mol Sci (2021) 22(5):2625. doi: 10.3390/ijms22052625 33807876 PMC7961376

[14] Drilon A Oxnard GR Tan DSW Loong HHF Johnson M Gainor J. Efficacy of Selpercatinib in Ret Fusion-Positive Non-Small-Cell Lung Cancer. New Engl J Med (2020) 383(9):813–24. doi: 10.1056/NEJMoa2005653 PMC7506467 32846060

[15] Pall G Gautschi O. Advances in the Treatment of Ret-Fusion-Positive Lung Cancer. Lung Cancer (Amsterdam Netherlands) (2021) 156:136–9. doi: 10.1016/j.lungcan.2021.04.017 33933276

[16] Illini O Hochmair MJ Fabikan H Weinlinger C Tufman A Swalduz A. Selpercatinib in Ret Fusion-Positive Non-Small-Cell Lung Cancer (Siren): A Retrospective Analysis of Patients Treated Through an Access Program. Ther Adv Med Oncol (2021) 13:17588359211019675. doi: 10.1177/17588359211019675 34178121 PMC8202258

[17] Subbiah V Velcheti V Tuch BB Ebata K Busaidy NL Cabanillas ME. Selective Ret Kinase Inhibition for Patients With Ret-Altered Cancers. Ann oncol: Off J Eur Soc Med Oncol (2018) 29(8):1869–76. doi: 10.1093/annonc/mdy137 PMC6096733 29912274

[18] Lin JJ Liu SV McCoach CE Zhu VW Tan AC Yoda S. Mechanisms of Resistance to Selective Ret Tyrosine Kinase Inhibitors in Ret Fusion-Positive Non-Small-Cell Lung Cancer. Ann oncol: Off J Eur Soc Med Oncol (2020) 31(12):1725–33. doi: 10.1016/j.annonc.2020.09.015 PMC9538591 33007380

[19] Subbiah V Shen T Terzyan SS Liu X Hu X Patel KP. Structural Basis of Acquired Resistance to Selpercatinib and Pralsetinib Mediated by Non-Gatekeeper Ret Mutations. Ann oncol: Off J Eur Soc Med Oncol (2021) 32(2):261–8. doi: 10.1016/j.annonc.2020.10.599 PMC7883646 33161056

[20] Della Corte CM Morgillo F. Rethinking Treatment for Ret-Altered Lung and Thyroid Cancers: Selpercatinib Approval by the Ema. ESMO Open (2021) 6(1):100041. doi: 10.1016/j.esmoop.2020.100041 33477006 PMC7820024

[21] Thein KZ Velcheti V Mooers BHM Wu J Subbiah V. Precision Therapy for Ret-Altered Cancers With Ret Inhibitors. Trends Cancer (2021) 7(12):1074–88. doi: 10.1016/j.trecan.2021.07.003 PMC8599646 34391699

[22] Fancelli S Caliman E Mazzoni F Brugia M Castiglione F Voltolini L. Chasing the Target: New Phenomena of Resistance to Novel Selective Ret Inhibitors in Lung Cancer. Updated Evidence and Future Perspectives. Cancers (2021) 13(5):1091. doi: 10.3390/cancers13051091 33806299 PMC7961559

[23] Gainor JF Curigliano G Kim DW Lee DH Besse B Baik CS. Pralsetinib for Ret Fusion-Positive Non-Small-Cell Lung Cancer (Arrow): A Multi-Cohort, Open-Label, Phase 1/2 Study. Lancet Oncol (2021) 22(7):959–69. doi: 10.1016/s1470-2045(21)00247-3 34118197

[24] Fda Approves Selpercatinib; Pralsetinib May Soon Follow. Cancer Discovery (2020) 10(7):Of1. doi: 10.1158/2159-8290.Cd-nb2020-052 32493697

[25] Wright KM. Fda Approves Pralsetinib for Treatment of Adults With Metastatic Ret Fusion-Positive Nsclc. Oncol (Williston Park NY) (2020) 34(10):406–. doi: 10.46883/onc.2020.3410.0406 33058106

[26] Sun F McCoach CE. Therapeutic Advances in the Management of Patients With Advanced Ret Fusion-Positive Non-Small Cell Lung Cancer. Curr Treat options Oncol (2021) 22(8):72. doi: 10.1007/s11864-021-00867-8 34165651

[27] Kim J Bradford D Larkins E Pai-Scherf LH Chatterjee S Mishra-Kalyani PS. Fda Approval Summary: Pralsetinib for the Treatment of Lung and Thyroid Cancers With Ret Gene Mutations or Fusions. Clin Cancer res: an Off J Am Assoc Cancer Res (2021) 27(20):5452–6. doi: 10.1158/1078-0432.Ccr-21-0967 34045295

[28] Horvath L Pircher A. Asco 2020 Non-Small Lung Cancer (Nsclc) Personal Highlights. Memo (2021) 14:66–9. doi: 10.1007/s12254-020-00673-2 PMC7804575 33456617

[29] Fu XY Dong XD Zeng L Ashby CR Jr. Chen ZS Cheng C. Pralsetinib for the Treatment of Non-Small Cell Lung Cancer. Drugs Today (Barcelona Spain: 1998) (2021) 57(9):559–69. doi: 10.1358/dot.2021.57.9.3306764 34586104

[30] Lei ZN Teng QX Gupta P Zhang W Narayanan S Yang DH. Cabozantinib Reverses Topotecan Resistance in Human Non-Small Cell Lung Cancer Nci-H460/Tpt10 Cell Line and Tumor Xenograft Model. Front Cell Dev Biol (2021) 9:640957. doi: 10.3389/fcell.2021.640957 33829017 PMC8019832

[31] Hayashi T Odintsov I Smith RS Ishizawa K Liu AJW Delasos L. Ret Inhibition in Novel Patient-Derived Models of Ret-Fusion Positive Lung Adenocarcinoma Reveals a Role for Myc Upregulation. Dis Models Mech (2020) 14(2):dmm047779. doi: 10.1242/dmm.047779 PMC7888717 33318047

[32] Wang Y Xu Y Wang X Sun C Guo Y Shao G. Ret Fusion in Advanced Non-Small-Cell Lung Cancer and Response to Cabozantinib: A Case Report. Medicine (2019) 98(3):e14120. doi: 10.1097/md.0000000000014120 30653139 PMC6370068

[33] Xing P Yang N Hu X Mu Y Wang S Guo Y. The Clinical Significance of Ret Gene Fusion Among Chinese Patients With Lung Cancer. Trans Cancer Res (2020) 9(10):6455–63. doi: 10.21037/tcr-20-754 PMC8797800 35117253

[34] Nokihara H Nishio M Yamamoto N Fujiwara Y Horinouchi H Kanda S. Phase 1 Study of Cabozantinib in Japanese Patients With Expansion Cohorts in Non-Small-Cell Lung Cancer. Clin Lung Cancer (2019) 20(3):e317–e28. doi: 10.1016/j.cllc.2018.12.018 30718102

[35] Roskoski R Jr. Sadeghi-Nejad A. Role of Ret Protein-Tyrosine Kinase Inhibitors in the Treatment Ret-Driven Thyroid and Lung Cancers. Pharmacol Res (2018) 128:1–17. doi: 10.1016/j.phrs.2017.12.021 29284153

[36] Ettinger DS Wood DE Aisner DL Akerley W Bauman JR Bharat A. Nccn Guidelines Insights: Non-Small Cell Lung Cancer, Version 2.2021. J Natl Compr Cancer Network: JNCCN (2021) 19(3):254–66. doi: 10.6004/jnccn.2021.0013 33668021

[37] Spanheimer PM Bashir A Lorenzen AW Beck AC Liao J Lizarraga IM. A Pilot Study of Preoperative Vandetanib on Markers of Proliferation and Apoptosis in Breast Cancer. Am J Clin Oncol (2021) 44(9):456–62. doi: 10.1097/coc.0000000000000845 PMC8387410 34190716

[38] Yoh K Seto T Satouchi M Nishio M Yamamoto N Murakami H. Final Survival Results for the Luret Phase Ii Study of Vandetanib in Previously Treated Patients With Ret-Rearranged Advanced Non-Small Cell Lung Cancer. Lung Cancer (Amsterdam Netherlands) (2021) 155:40–5. doi: 10.1016/j.lungcan.2021.03.002 33725547

[39] Wang M Naganna N Sintim HO. Identification of Nicotinamide Aminonaphthyridine Compounds as Potent Ret Kinase Inhibitors and Antitumor Activities Against Ret Rearranged Lung Adenocarcinoma. Bioorganic Chem (2019) 90:103052. doi: 10.1016/j.bioorg.2019.103052 31226468

[40] Dudnik E Bshara E Grubstein A Fridel L Shochat T Roisman LC. Rare Targetable Drivers (Rtds) in Non-Small Cell Lung Cancer (Nsclc): Outcomes With Immune Check-Point Inhibitors (Icpi). Lung Cancer (Amsterdam Netherlands) (2018) 124:117–24. doi: 10.1016/j.lungcan.2018.07.044 30268448

[41] Dantoing E Piton N Salaün M Thiberville L Guisier F. Anti-Pd1/Pd-L1 Immunotherapy for Non-Small Cell Lung Cancer With Actionable Oncogenic Driver Mutations. Int J Mol Sci (2021) 22(12):6288. doi: 10.3390/ijms22126288 34208111 PMC8230861

[42] Rosen EY Johnson ML Clifford SE Somwar R Kherani JF Son J. Overcoming Met-Dependent Resistance to Selective Ret Inhibition in Patients With Ret Fusion-Positive Lung Cancer by Combining Selpercatinib With Crizotinib. Clin Cancer Res an Off J Am Assoc Cancer Res (2021) 27(1):34–42. doi: 10.1158/1078-0432.Ccr-20-2278 PMC8009613 33082208

[43] Lee J Ku BM Shim JH La Choi Y Sun JM Lee SH. Characteristics and Outcomes of Ret-Rearranged Korean Non-Small Cell Lung Cancer Patients in Real-World Practice. Jpn J Clin Oncol (2020) 50(5):594–601. doi: 10.1093/jjco/hyaa019 32083304

[44] Mazieres J Drilon A Lusque A Mhanna L Cortot AB Mezquita L. Immune Checkpoint Inhibitors for Patients With Advanced Lung Cancer and Oncogenic Driver Alterations: Results From the Immunotarget Registry. Ann oncol: Off J Eur Soc Med Oncol (2019) 30(8):1321–8. doi: 10.1093/annonc/mdz167 PMC7389252 31125062

[45] Hanna NH Robinson AG Temin S Baker S Jr. Brahmer JR Ellis PM. Therapy for Stage Iv Non-Small-Cell Lung Cancer With Driver Alterations: Asco and Oh (Cco) Joint Guideline Update. J Clin oncol: Off J Am Soc Clin Oncol (2021) 39(9):1040–91. doi: 10.1200/jco.20.03570 33591844

[46] Hida T Velcheti V Reckamp KL Nokihara H Sachdev P Kubota T. A Phase 2 Study of Lenvatinib in Patients With Ret Fusion-Positive Lung Adenocarcinoma. Lung Cancer (Amsterdam Netherlands) (2019) 138:124–30. doi: 10.1016/j.lungcan.2019.09.011 31710864

[47] Takeuchi S Murayama T Yoshimura K Kawakami T Takahara S Imai Y. Phase I/Ii Study of Alectinib in Lung Cancer With Ret Fusion Gene: Study Protocol. J Med invest: JMI (2017) 64(3.4):317–20. doi: 10.2152/jmi.64.317 28955006

[48] Horiike A Takeuchi K Uenami T Kawano Y Tanimoto A Kaburaki K. Sorafenib Treatment for Patients With Ret Fusion-Positive Non-Small Cell Lung Cancer. Lung Cancer (Amsterdam Netherlands) (2016) 93:43–6. doi: 10.1016/j.lungcan.2015.12.011 26898613

[49] Ferrara R Auger N Auclin E Besse B. Clinical and Translational Implications of Ret Rearrangements in Non-Small Cell Lung Cancer. J Thorac oncol: Off Publ Int Assoc Study Lung Cancer (2018) 13(1):27–45. doi: 10.1016/j.jtho.2017.10.021 29128428

[50] Lin C Wang S Xie W Zheng R Gan Y Chang J. Apatinib Inhibits Cellular Invasion and Migration by Fusion Kinase Kif5b-Ret *Via* Suppressing Ret/Src Signaling Pathway. Oncotarget (2016) 7(37):59236–44. doi: 10.18632/oncotarget.10985 PMC5312308 27494860

[51] Drilon A Bergagnini I Delasos L Sabari J Woo KM Plodkowski A. Clinical Outcomes With Pemetrexed-Based Systemic Therapies in Ret-Rearranged Lung Cancers. Ann oncol: Off J Eur Soc Med Oncol (2016) 27(7):1286–91. doi: 10.1093/annonc/mdw163 PMC4922319 27056998

[52] Berghoff AS Bellosillo B Caux C de Langen A Mazieres J Normanno N. Immune Checkpoint Inhibitor Treatment in Patients With Oncogene- Addicted Non-Small Cell Lung Cancer (Nsclc): Summary of a Multidisciplinary Round-Table Discussion. ESMO Open (2019) 4(3):e000498. doi: 10.1136/esmoopen-2019-000498 31297240 PMC6586213

[53] Hess LM Han Y Zhu YE Bhandari NR Sireci A. Characteristics and Outcomes of Patients With Ret-Fusion Positive Non-Small Lung Cancer in Real-World Practice in the United States. BMC Cancer (2021) 21(1):28. doi: 10.1186/s12885-020-07714-3 33402119 PMC7786962

[54] Codony-Servat J García-Roman S Molina-Vila M Bertran-Alamillo J Viteri S d'Hondt E. Anti-Epidermal Growth Factor Vaccine Antibodies Increase the Antitumor Activity of Kinase Inhibitors in Alk and Ret Rearranged Lung Cancer Cells. Trans Oncol (2021) 14(1):100887. doi: 10.1016/j.tranon.2020.100887 PMC7591385 33129112

[55] Solomon BJ Tan L Lin JJ Wong SQ Hollizeck S Ebata K. Ret Solvent Front Mutations Mediate Acquired Resistance to Selective Ret Inhibition In Ret-Driven Malignancies. J Thorac Oncol Off Publ Int Assoc Study Lung Cancer (2020) 15(4):541–9. doi: 10.1016/j.jtho.2020.01.006 PMC7430178 31988000

[56] Yoda S Lin JJ Lawrence MS Burke BJ Friboulet L Langenbucher A. Sequential Alk Inhibitors Can Select for Lorlatinib-Resistant Compound Alk Mutations in Alk-Positive Lung Cancer. Cancer Discov (2018) 8(6):714–29. doi: 10.1158/2159-8290.Cd-17-1256 PMC5984716 29650534

[57] Nelson-Taylor SK Le AT Yoo M Schubert L Mishall KM Doak A. Resistance to Ret-Inhibition in Ret-Rearranged Nsclc Is Mediated by Reactivation of Ras/Mapk Signaling. Mol Cancer Ther (2017) 16(8):1623–33. doi: 10.1158/1535-7163.Mct-17-0008 PMC5544556 28500237

[58] Tan L Solomon BJ. Defining Resistance Mechanisms to Selective Ret Tyrosine Kinase Inhibitors in Ret Fusion-Positive Non-Small-Cell Lung Cancer. Ann Oncol Off J Eur Soc Med Oncol (2020) 31(12):1599–600. doi: 10.1016/j.annonc.2020.10.002 33045324

[59] Piotrowska Z Isozaki H Lennerz JK Gainor JF Lennes IT Zhu VW. Landscape of Acquired Resistance to Osimertinib in Egfr-Mutant Nsclc and Clinical Validation of Combined Egfr and Ret Inhibition With Osimertinib and Blu-667 for Acquired Ret Fusion. Cancer Discov (2018) 8(12):1529–39. doi: 10.1158/2159-8290.Cd-18-1022 PMC6279502 30257958

[60] Suda K Mitsudomi T. Emerging Oncogenic Fusions Other Than Alk, Ros1, Ret, and Ntrk in Nsclc and the Role of Fusions as Resistance Mechanisms to Targeted Therapy. Trans Lung Cancer Res (2020) 9(6):2618–28. doi: 10.21037/tlcr-20-186 PMC7815361 33489822

